# Systematic review and meta-analysis of the prevention of internalizing disorders in early childhood

**DOI:** 10.3389/fpsyg.2023.1061825

**Published:** 2023-12-14

**Authors:** Brigid Bolton, Rosanna Mary Rooney, Anya Hughes, Amber Hopkins, Vincent Oreste Mancini

**Affiliations:** ^1^Psychology Department, Curtin School of Population Health, Faculty of Health Sciences, Bentley, WA, Australia; ^2^Discipline of Psychology, School of Population Health, Curtin University, Bentley, WA, Australia; ^3^Human Development and Community Wellbeing, Telethon Kids Institute, Nedlands, WA, Australia; ^4^Division of Paediatrics, UWA Medical School, University of Western Australia, Perth, WA, Australia; ^5^The Fathering Project, Sydney, NSW, Australia

**Keywords:** meta-analysis, anxiety, depression, early childhood, internalizing, prevention

## Abstract

**Introduction:**

Internalizing problems comprise a significant amount of the mental health difficulties experienced during childhood. Implementing prevention programs during early childhood may prevent internalizing problems. The present systematic review and meta-analysis aimed to evaluate the effect of both targeted and universal prevention programs in preventing internalizing problems for children aged 3- to 5-years and their parents.

**Methods:**

PsycINFO, Embase, and MEDLINE were systematically searched, and 17 randomized control trials, consisting of 3,381 children, met eligibility criteria. There were seven universal prevention programs, and 10 targeted prevention programs. Four prevention programs were delivered to children, 10 prevention programs were delivered to parents/caregivers, and three prevention programs were delivered to both parents and children.

**Results:**

Prevention programs led to significantly fewer internalizing problems at 6- and 7-month post-intervention (*n* = 7, *p* = 0.02, CI −0.69, 0.06) with a small-to-moderate effect size (*g* = −0.38), however, not at post-intervention or at 12-month follow up.

**Discussion:**

Overall, findings suggest that there may be value in ongoing development and evaluation of prevention programs for internalizing problems, as they improve social and emotional wellbeing in students and reduce internalizing difficulties within the 6- to 7-month timeframe following prevention programs.

**Systematic review registration:**

PROSPERO: CRD42021261323.

## Introduction

Internalizing problems are a leading contributor to health burden amongst young people globally, with mental disorders affecting one in five children (Bitsko et al., 2022) and estimates of one in four during the COVID-19 pandemic (Australian Institute of Health Welfare, 2020). Internalizing problems encompass a range of difficulties characterized by emotional distress and symptoms associated with anxiety and depression (Wergeland et al., 2021). Internalizing problems can be a combination of cognitive, physiological, and behavioral symptoms and are associated with a significant impact on functioning (Kertz et al., 2019).

There is significant intersection between depression and anxiety symptoms, and they can be clustered within the construct of internalizing problems (Lee and Vaillancourt, 2020). Anxiety is the second most common disorder in childhood and affects up to 9% of young children (Ghandour et al., 2019). While depressive disorders are less common, affecting approximately 3% of children (Doering et al., 2022), there is evidence that there is considerable stability of internalizing problems from 3 years of age (Hatoum et al., 2018). Further, earlier onset of depression and anxiety is attributed to a worse clinical outcome over the lifespan (Finsaas et al., 2020), as both depression and anxiety can be chronic and recurring. When considering the current COVID-19 pandemic, emerging evidence has shown an effect of the pandemic on the psychological health of parents and children (Crescentini et al., 2020). The pandemic has had a significant psychosocial impact on young people and is thought to contribute to higher instances of anxiety and depression (Duan et al., 2020).

The impact of internalizing problems is wide-ranging and encompasses emotional, social, and economic costs, even in younger children (Pedersen et al., 2019). The third and fourth leading causes of the burden of disease for children in Australia are anxiety-related problems and psychological developmental problems, respectively (Australian Institute of Health Welfare, 2020). Internalizing problems have a pervasive impact on numerous areas of life such as adaptive functioning, relationships, academic pursuits, school engagement, and they can further impact mental health and relationships as children grow older (Caldwell et al., 2021). If internalizing disorders are not treated effectively, they can have a significant negative effect on development, and children's long-term capacity to live productive, healthy, and fulfilling lives (Chatterton et al., 2020). In Australia, the annual expense of mental health problems to the economy is estimated to be $70 billion, and large savings are theorized if mental illness can be prevented through early intervention (Productivity Commission, 2020).

Prevention in early childhood can have a significant impact developmentally, as opposed to later school or adult intervention (Bierman et al., 2021). Due to mental health difficulties in early life having a significant impact on future health, it is pertinent to build the foundations of social and emotional learning during early development. For example, the British National Child Development Study found that internalizing problems from ages in early childhood could be predictive of higher mortality by age 45 (Jokela et al., 2009; Eurenius et al., 2021). The continued burden of mental health may suggest that current treatment may not be significantly reducing the effect and prevalence of internalizing problems. Prevention programs in early childhood can reduce symptoms and delay the onset of internalizing symptoms (Stockings et al., 2016; Loevaas et al., 2020). Utilizing a preventative approach during early childhood can be more effective as patterns of behavior have not already been established (Davey and McGorry, 2019).

Prevention programs generally comprise either universal or targeted prevention approaches. Universal prevention approaches are delivered to all individuals in a population (e.g., a classroom; Bernaras et al., 2019). Universal prevention programs can be beneficial as they can target a large population, reduce stigma within the population and individual children do not feel like they are targeted (Baughman et al., 2020). They can also minimize the risk of overlooking students which is beneficial as children with internalizing problems can exhibit compliant and non-disruptive behavior (Baughman et al., 2020). In contrast, targeted or selective approaches focus on children at risk of developing a psychological disorder (Bernaras et al., 2019). Growing research has shown the value of well-designed prevention programs across both levels in preventing, delaying onset, and reducing internalizing problems in children (Caldwell et al., 2019).

Prevention programs are a proactive approach to preventing psychopathology and support children's development of social-emotional competence (Lakes et al., 2019). Lower social-emotional competence and the skills associated are a predictor of internalizing behaviors (Huber et al., 2019). Social-emotional competence encompasses emotional, cognitive, and behavioral areas of development, including awareness of and regulation of emotions, emotional literacy, perspective-taking, and problem-solving skills (Green et al., 2021). Cognitive, emotional, and behavioral skills are important for prosocial behavior and the prevention of internalizing problems (Eklund et al., 2018).

Effective social and emotional prevention programs have been available for older children in primary school and lower high school for some time, with literature supporting them (Ishikawa et al., 2019). However, social and emotional skill development is considered to be beneficial in early childhood to enhance social-emotional competencies (Aksoy, 2019). Early childhood prevention programs for internalizing problems are relatively new, and research is still developing (Baughman et al., 2020). Few preventative programs are both accessible and suitable for children younger than 5 years old (Forbes et al., 2019).

While reviews and meta-analyses on anxiety and depression prevention initiatives in children have been completed, there are still gaps in the literature. In particular, a large network meta-analysis focused on children aged 4- to 18-years, however only in school-based settings (Caldwell et al., 2019). Caldwell et al. (2019) review did not include online, or community-based programs and they also did not assess prevention programs for 3-year-old children. Further systematic reviews and meta-analyses have been conducted on the prevention of depression and anxiety in young people, both children and adolescents, however, most have focused on children over 5-years of age and have not addressed prevention outside of schools (Johnstone et al., 2018).

Baughman et al. (2020) reviewed programs within schools and the community for children aged 4–6 years and their parents. They completed searches within the Australian mental health promotion website *BeYou* and the *Cochrane Library* database. The review concluded that prevention efforts earlier in childhood were needed to reduce the burden associated with internalizing problems, however, they were only able to identify six programs that could be delivered as prevention programs. They further identified that skills training in cognitive and social-emotional areas is effective in reducing internalizing problems. While the programs they reviewed showed favorable support for prevention in early childhood, more rigorous studies were needed that would involve longer-term randomized controlled trials. Baughman et al. (2020) review gave an excellent picture of the programs in place for young children, and it is thought that a meta-analysis will provide further clarity on the effectiveness of current interventions.

Research findings suggest that children from 3 years of age are well placed to receive social and emotional education (Ardoin and Bowers, 2020; Blewitt et al., 2021). However, most prevention programs and reviews have focused on children within the school system and over the age of four or five (Caldwell et al., 2019). For younger children, community-based interventions focusing on parenting or social-emotional learning may be well suited (e.g., child health centers, playgroups etc.). Research has also led to recommendations that interventions delivered to parents may positively support children's internalizing problems (Baughman et al., 2020). During the COVID-19 pandemic, it also became apparent how beneficial online support and programs can be. Therefore, reviewing the effectiveness of programs across the community for children aged 3- to 5-years and their parents is required.

While there are few internalizing disorder prevention programs created for early childhood; the programs available appear to show promising results. To date, no studies have systematically reviewed social and emotional programs specifically addressing children aged 3- to 5-years. The current study aims to investigate the effectiveness of social and emotional programs in the prevention of internalizing symptoms in children aged 3- to 5-years. The study will examine data from prevention programs to provide more conclusive results regarding the effectiveness of the programs.

This paper conducts a systematic review and meta-analysis of randomized control trials evaluating programs for use with children aged 3- to 5-years and their parents. To facilitate the further adoption and creation of programs, the systematic review will address the following research questions:

1. Are social and emotional programs effective for the prevention of internalizing disorders in early childhood (3- to 5-years)?
2. Which characteristics of the featured programs are related to the effectiveness of the programs in reducing internalizing disorders?

## Materials and methods

### Protocol and registration

The study's protocol was prospectively published and registered with the International Prospective Register of Systematic Reviews (PROSPERO) before screening studies for inclusion and was allocated the registration number CRD42021261323. An amendment was reported, changing the eligibility criteria to only include randomized controlled trials. The preferred reporting items for systematic reviews and meta-analyses (PRISMA) guidelines were consulted to perform the literature review (Page et al., 2021), (see Supplementary material).

### Eligibility criteria

#### Types of studies

The review considered experimental studies where they were (a) published from 2000 to 2021, to ensure that the programs were still feasible to continue to be executed, (b) written in English, due to language constraints of the reviewers, (c) used a randomized control trial (RCT) methodology, due to the methodology being considered the gold standard, and (d) published in a peer-reviewed scientific journal.

#### Participants

Population: The review considered studies that included children with a mean age between 3 and 5 years. Studies focusing on children with externalizing difficulties, developmental disabilities, or that were treating a specific disorder were not included.

#### Inclusion criteria

Intervention: The review included universal, and targeted interventions (i.e., children at risk) that focused on preventing internalizing problems. Studies were eligible if they included psychological, psychosocial, or educational interventions that were implemented to either children or parents. If the study's intervention also focused on parents but assessed the intervention's outcome on children, it was still included. The review only focused and reported on the specific outcomes for children. Comparators: Studies were included when the intervention group was compared to a control group. Randomized control trial methodologies were included due to being considered more rigorous and were thought to contribute to the quality of studies Outcomes: Internalizing symptoms were the primary outcome measure for this review, e.g., the Strengths and Difficulties Questionnaire (Goodman, 1997), Behavior Assessment System for Children (Reynolds, 2010), and the Preschool Anxiety Scale (Edwards et al., 2010). Exclusion criteria: Studies were excluded if they were duplicates or used duplicate samples and data. Duplicates were be identified and removed based on authors or sample populations and/or sample data. Literature reviews and meta-analyses were also excluded. Follow up: Some studies provided follow up data (i.e., 6 and 12 months following the intervention). Follow up data was extracted if available, however the control groups were required to have remained the same throughout the study and not have received any intervention.

### Information sources and search strategy

The full electronic search was completed on August 12th, 2021. Three electronic databases (PsycINFO, Embase, and MEDLINE) were identified and searched for articles. The databases were selected due to the social and emotional programs being multidisciplinary and that these databases allow searches to be refined to participants' age ranges. Databases were searched using a combination of terms (full search terms are provided in Supplementary material).

### Data collection and analysis

#### Selection of studies

Studies for inclusion were imported to Endnote X9 software (Clarivate Analytics, 2018) and duplicates were removed. An eligibility assessment was performed in a standardized manner by one reviewer. Titles and abstracts from the initial search were reviewed to determine the relevance of the articles. Studies from the initial search were excluded if the title and abstract did not meet the eligibility criteria. The remainder of the studies were read, reviewed, and selected for inclusion if they met the standard inclusion criteria. A second reviewer then utilized ASReview software (van de Schoot et al., 2021), reviewed ten percent of the initial search, and then had machine learning complete the review of articles. Reviewers resolved any differences through consensus. Additional reference mining was then also completed, the selected study's reference lists and citations were reviewed through a hand search.

#### Data screening

**Figure 1:**
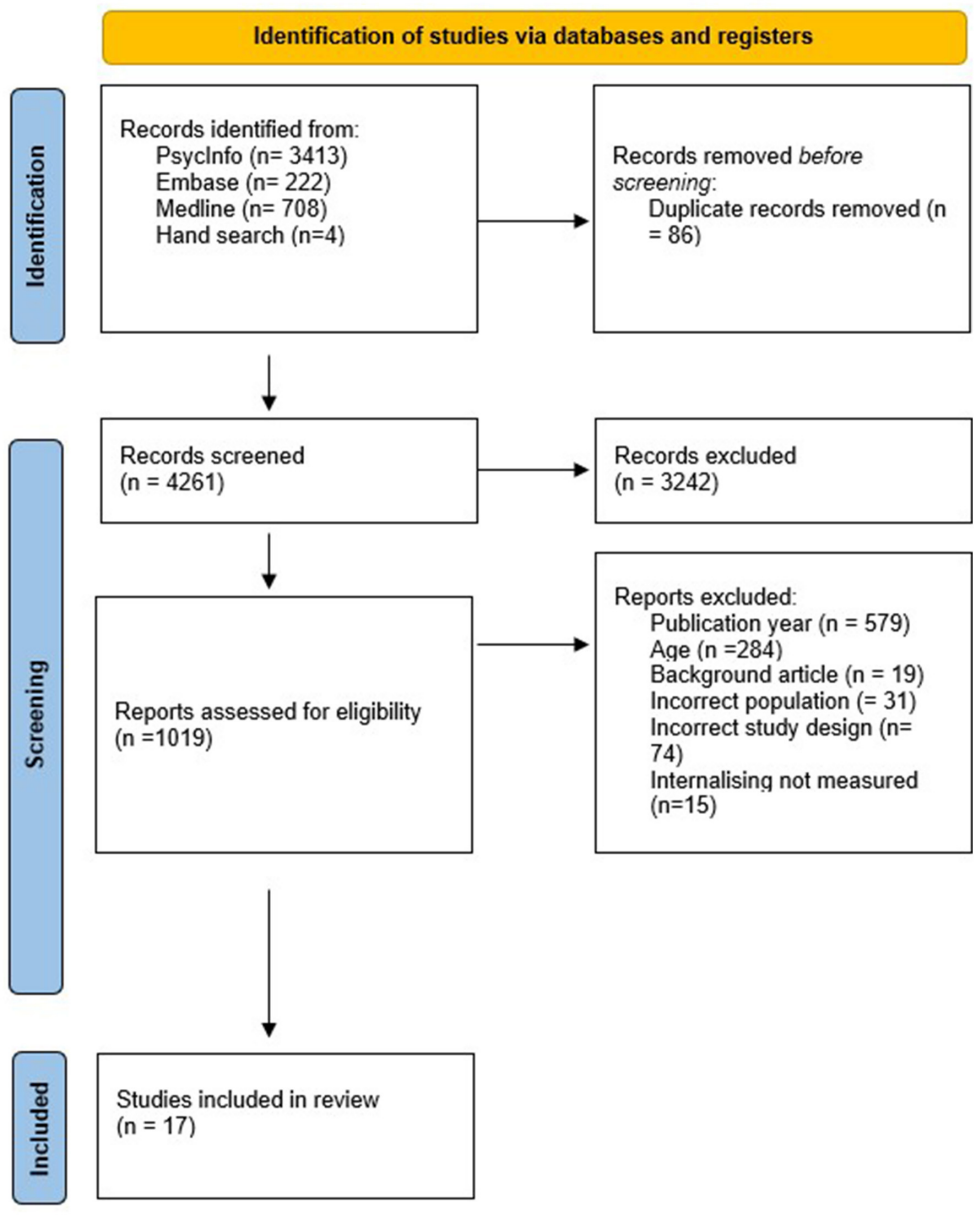
Identification and selection of studies for inclusion in the meta-analysis. Adapted from The PRISMA Statement (Page et al., 2021).

The screening process of studies is displayed in a “Flow of Studies” diagram as depicted below in Figure 1 as per the PRISMA guidelines (Page et al., 2021).

#### Data extraction

One researcher independently obtained details from the included RCTs. The information that was extracted included the study, country, intervention program, population, sample size, participants' ages, number of sessions, the mode of delivery, the outcome measure(s), and the measure intervals (see Table 1).

**Table 1:** Summary of the study characteristics of the included studies (ordered by author).

**Study**	**Country**	**Prevention type**	**Intervention program**	**Population**	***N***	**Age range, (M/SD)**	**Sessions**	**Mode of delivery**	**Outcome measure**	**Measure times**
Anticich et al. (2013)	Australia	Universal	Fun FRIENDS	School Students	488	4–7 (5.42/0.67)	10	Children: Teachers delivering to children in classrooms	Preschool anxiety scale	Pre, post, 12 mo FO
Bayer et al. (2018)	Australia	Targeted	Cool Little Kids	Inhibited preschool children	545	4 years (4/0.4)	6	Parents: Manualised parenting group sessions	Strengths and difficulties questionnaire	Pre, 12 mo FO
Carta et al. (2013)	America	Targeted	Cellular Phone Enhanced Planned Activities Training	At risk for child maltreatment	371	3.5–5.5 (4.56/0.57)	5	Parents: Manualised parenting group sessions	Behavior assessment system for children	Pre, post, 6 mo FO
Cartwright-Hatton et al. (2018)	England	Targeted	CBT workshop	Parents with an anxiety disorder	100	3–9 (5.49)	1	Parents: One day CBT workshop	Spence children's anxiety scale	Pre, 3 mo FO, 12 mo FO
Chronis-Tuscano et al. (2015)	America	Targeted	Turtle Program	Inhibited children	30	3.5–5 (6.5)	8	Parents: Parent Child Interaction Therapy Children: Social Skills Facilitated Play	Child behavior checklist	Pre, post
Dadds and Roth (2008)	Australia	Universal	Reach for Resilience	Preschool students	734	3–6 (4.77/0.47)	6	Parents: CBT Training program	Social competence and behavior evaluation	Post, and 7 mo FO
Domitrovich et al. (2019)	America	Universal	PATHS Program	School students	246	3–5 (4.28/0.49)	30	Children: Teachers delivering lessons	Preschool and kindergarten behavior scales	Pre, post
Edrissi et al. (2019)	Iran	Targeted	Tuning into Kids	Anxious preschool children	56	4–6 (4.4/0.75)	8	Parents: group training program	Preschool anxiety scale	Pre, post, 6 mo FO
Eninger et al. (2021)	Sweden	Universal	PATHS Program	Preschool children	285	4–5 (4.8/0.6)	33	Children: Teachers delivering lessons	Preschool and kindergarten behavior scales	Pre, post
Fishbein et al. (2016)	America	Universal	PATHS Program	Schools in high poverty neighborhoods	327	Kindergarten age	44	Children: Teachers delivering to children in classrooms	Teacher observation of classroom adaptation- revised	Pre, post
Hahlweg et al. (2010)	Germany	Universal	Triple P Positive Parenting Program	Preschools	282	2.6–6 (4.5/1)	4	Parents: Training	Child behavior checklist	Pre, post, 12 mo FO, 24 mo FO
Kennedy et al. (2009)	Australia	Targeted	Cool Kids	Behaviorally inhibited children and parents an anxiety disorder	71	3–4.8 (3.9/0.58)	8	Parents: Manualised parenting group sessions	Preschool anxiety scale revised	Pre, 6 mo FO
Lau et al. (2017)	Australia	Targeted	Cool Little Kids and modified Social Skills Facilitated Play	Behavioral inhibited children and parents experiencing high emotional distress	72	3–5.4 (4.34)	6	Parents: Manualised parenting group sessions Children: Social Skills training	Preschool anxiety scale revised	Pre, 6 mo FO
Morgan et al. (2017)	Australia	Targeted	Cool Little Kids	Children with an inhibited temperament	422	3–6 (4.8/1)	8	Parents: Online training- CBT	Strengths and difficulties questionnaire	Pre, 3 mo,FO, 6 mo FO
N'zi et al. (2016)	America	Targeted	Child Directed Interaction Training	Children living in kinship care with their grandmothers	14	2–7 (5.42/1.17)	8	Caregivers/Grandmothers: Sessions at a local community library	Child behavior checklist	Pre, post
Pahl and Barrett (2010)	Australia	Universal	The Fun FRIENDS Program	Preschool students attending preschool	263	4–6 (4.56/0.51)	9	Children: Teachers delivering lessons to classrooms Parents: information and psychoeducation sessions	Preschool anxiety scale	Pre, post and 12 mo fo
Rapee et al. (2005)	Australia	Targeted	Cool Little Kids	Behaviorally inhibited children	146	3–5 (3.9/0.42)	6	Parents: Manualised parenting group sessions	Anxiety and related disorders interview schedule	Pre, 12 mo FO

Mo, month; FO, follow-up.

Data extracted for analysis included means, standard deviations, and sample sizes for intervention and control group at pre-intervention, post-intervention, and follow-up on reliable and valid outcome/symptom rating scales for depression, anxiety, and internalizing symptoms (see Supplementary material). If multiple internalizing measures were used in studies, the measure that was selected in the study to specifically assess internalizing symptoms and was most representative of internalizing symptoms were used. When studies reported mothers and fathers separately, data were assessed separately. When studies utilized multiple comparison conditions, the conditions were separated, and both were compared to the control condition. It was ensured that intervention groups were not included twice in a synthesized effect size. If studies utilized an intervention group, an active control, and a waitlist control, the intervention and active control data were assessed separately. When data was unable to be extracted, the reviewer contacted the article's corresponding author to attain the data, if data was unable to be attained, the studies were removed from the meta-analysis (see Supplementary material). Due to a short timeframe to run the data-analysis, some data was attained from contacted authors, however, it was not within the timeframe of the data-analysis and was unable to be included.

#### Assessment of study quality and risk of bias

Quality and risk of bias was assessed using the Revised Cochrane risk-of-bias tool (RoB 2; Higgins and Thomas, 2019). Risk of bias was assessed by one researcher. Risk of bias was reported separately for each of the five criteria: the randomization process, deviations from the intended interventions, missing outcome data, measurement of the outcome, and selection of the reported result. Domains were scored as (1) low risk, (2) some concerns, and (3) high risk.

#### Statistical analysis

##### Data synthesis

All data were synthesized using the statistical software program Jamovi and the Meta-Analysis for JAMOVI, R (MAJOR) Package version 1.2.0. (Hamilton, 2019; The Jamovi Project, 2021). A meta-analysis was completed on data from all studies to examine the intervention effects for internalizing problems as a complete construct. Two additional analyses were planned to assess if the interventions had a continuing effect after follow-up assessment, 6–7 months, and 12 months.

##### Effect size calculations

Effect size was calculated utilizing Hedges *g*, calculated as:

$g=\frac{M_{1}-M_{2}}{SD_{pooled}}$. Hedge's *g* was utilized due to the small number of studies included, as it is more conservative. Standardized mean differences were used (the standardized mean difference between the two groups at post-treatment) as it accounted for the variability in measurements used for the study outcomes and it includes an adjustment to address small sample sizes (Hedges and Olkin, 2014). A 95% confidence interval was reported. Hedge's *g* was interpreted according to Cohen's guidelines of 0.2, 0.5, and 0.8 referring to small, moderate, and large effect sizes, respectively (Cohen, 2013).

##### Aggregation of effect sizes

The aggregate effect sizes were estimated using the random effects model, which makes the assumption that true effect size varies in each study, therefore the studies in the analysis represent a random sample of effect-sizes. The approach allows for the estimation of differences in the effects of studies that are not attributed to error. The estimated heterogeneity is then used to support the accurate weighting of individual studies when estimating the aggregation of effect size (DerSimonian and Kacker, 2007).

Statistical heterogeneity was determined through the Q statistic and *I*^2^ statistic. To quantify the heterogeneity in the pooled estimates *I*^2^statistic index was used, where heterogeneity was classified as low, moderate, or high with an *I*^2^statistic value of 25, 50, and 75%, respectively (Higgins and Thomas, 2019).

##### Moderator analyses

Moderator analysis should only be utilized when there is significant variability across effect sizes, which may suggest the likelihood of a moderator. The study used *I*^2^statistic to indicate if moderator analyses should be conducted. Two moderators were planned, assessing prevention type (i.e., targeted, and universal prevention), and who the prevention was provided to (i.e., caregiver[s], or child).

##### Funnel plot asymmetry

To further assess for publication bias, the funnel plot was examined. The funnel plot plots the study's effect sizes against standard error, assuming that effects from larger studies are generally more consistent (Egger et al., 1997). Egger et al. (1997) regression test was proposed to be used to test funnel plot asymmetry.

## Results

### Description of studies

#### Study characteristics

Of the seventeen studies identified, a total number of 1,813 intervention and 1,568 control participants were included. Sample sizes of the included studies varied considerably from between 14 participants (N'zi et al., 2016), and 734 (Dadds and Roth, 2008), with a median of 263 participants. Of the seventeen studies, eight studies were from Australia, five studies were from USA, and the remaining studies were from Germany, Iran, Sweden, and England.

#### Prevention type

There were seven universal prevention programs, and ten targeted prevention programs. All universal prevention programs took place within a school setting. Of the studies implementing targeted preventions, six studies focused on populations of inhibited preschool children (Rapee et al., 2005; Kennedy et al., 2009; Chronis-Tuscano et al., 2015; Lau et al., 2017; Morgan et al., 2017; Bayer et al., 2018), one study focused on low-income families at risk of maltreatment (Carta et al., 2013), one study focused on children with parents that have a diagnosed anxiety disorder (Cartwright-Hatton et al., 2018), one study focused on children with elevated anxiety symptoms (Edrissi et al., 2019), and one study focused on children living in kinship care (N'zi et al., 2016).

#### Randomization

The randomization in studies varied in terms of whether it occurred at the school (Dadds and Roth, 2008; Hahlweg et al., 2010; Anticich et al., 2013; Fishbein et al., 2016; Eninger et al., 2021; 29%), class (13%; Domitrovich et al., 2007; Pahl and Barrett, 2010), or individual level (58%). All studies that utilized a targeted prevention method utilized individual randomization.

#### Control groups

Six studies utilized usual care as their control group and had no intervention/treatment as usual (Rapee et al., 2005; Domitrovich et al., 2007; Dadds and Roth, 2008; Hahlweg et al., 2010; Bayer et al., 2018; Cartwright-Hatton et al., 2018), nine studies utilized waitlist control groups, where the control group underwent the intervention at a later date (Kennedy et al., 2009; Pahl and Barrett, 2010; Carta et al., 2013; Chronis-Tuscano et al., 2015; N'zi et al., 2016; Lau et al., 2017; Morgan et al., 2017; Edrissi et al., 2019; Eninger et al., 2021), and one study utilized an unspecified attention control (Fishbein et al., 2016). It is noted that Anticich et al. (2013) had an intervention group, an active comparison group, and a waitlist control; the active comparison and intervention groups were separated independently in the statistical analysis.

#### Prevention programs

Four prevention programs were delivered to children, ten prevention programs were delivered to parents/caregivers, and three prevention programs were delivered to both parents and children. Most intervention programs identified in the review utilized cognitive behavior therapy (CBT; 53%). Other studies fell under the umbrella of parent training, that is, Planned Activities Training (Carta et al., 2013), Parent Child Interaction therapy (Chronis-Tuscano et al., 2015; N'zi et al., 2016), Emotion-Focused Parenting (Edrissi et al., 2019), and Triple P Positive Parenting Program (Hahlweg et al., 2010). Three studies also utilized the PATHS program which is based on the affective-behavior-cognitive-dynamic model (Domitrovich et al., 2007; Fishbein et al., 2016; Eninger et al., 2021).

#### Program format and mode of delivery

Eight of the prevention programs were facilitated by psychologists, three were facilitated by post-graduate psychology trainees, four programs were facilitated by teachers, one program was presented online and was parent-led, and one program was facilitated by research staff with a bachelor's degree. The majority of programs were conducted in a group setting (82%), two targeted (12%) programs were provided individually (Carta et al., 2013; N'zi et al., 2016) focusing on parenting, and one program (6%; was conducted individually to parents completing an online program (Morgan et al., 2017).

#### Program sessions

The length of the programs ranged from a one-day session to 44 sessions, with most programs (53%) being delivered in between six and eight sessions (median = 8). Most studies (76%) ran the majority of sessions weekly, with two studies running sessions fortnightly, one running sessions biweekly, and one running a day workshop.

#### Outcome measures

Of the studies identified in the systematic review, the majority used measures that assessed internalizing problems as a construct (53%), the remaining studies specifically assessed anxiety symptoms (47%), no studies specifically assessed symptoms of depression in children. Of the studies that assessed internalizing problems, two studies used the Strengths and Difficulties Questionnaire (Goodman, 1997), one study used the Behavior Assessment System for Children (Reynolds, 2010), three studies used the Child Behavior Checklist (Achenbach, 1999), two studies used the Preschool and Kindergarten Behavior Scales (Merrell, 2002), and one study used the Teacher Observation of Classroom Adaptation Revised (Werthamer-Larsson et al., 1989). Of the studies that assessed anxiety symptoms, five studies utilized the Preschool Anxiety Scale (Edwards et al., 2010), one study used the Anxiety and Related Disorders Interview Schedule (Brown and Barlow, 2014), one study used the Spence Children's Anxiety Scale (Spence, 1997), and one used the Social Competence and Behavior Evaluation (LaFreniere and Dumas, 1996).

#### Follow-up

Eleven studies identified in the systematic review reported pre- and post-data for the control and intervention groups, two studies reported 3-month follow-up data, one study reported 7-month follow-up data, four studies reported 6-month follow-up data, seven studies reported 12-month follow-up data, and one study reported 24-month follow-up data.

#### Risk of bias

**Figure 2:**
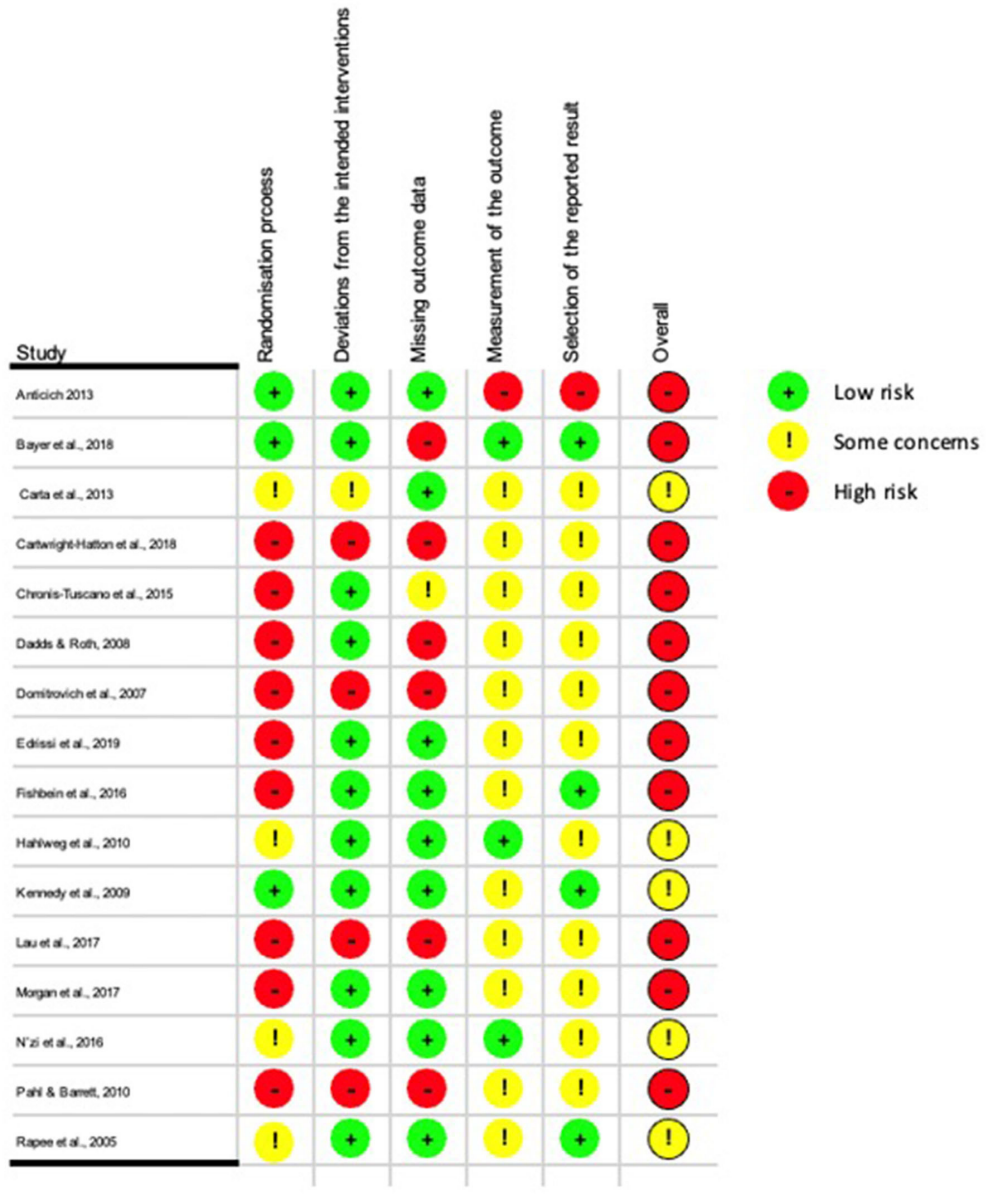
Methodological quality summary: judgements about each methodological quality item for each included study.

**Figure 3:**
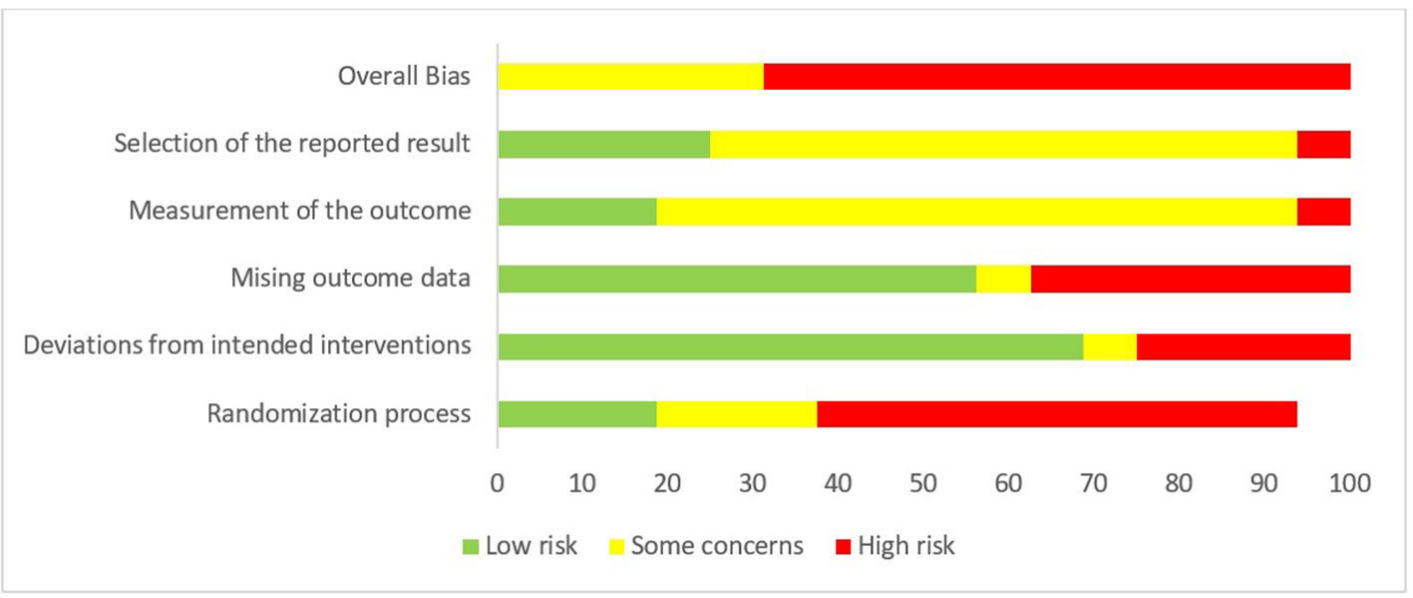
Methodological quality summary: judgments about each methodological quality item for each included study—as presented as percentages across all included studies.

The 17 RCTs were evaluated using the RoB2 tool. The quality of the methodology of the studies reported varied substantially (see Figure 2 for study quality ratings). Overall, five studies had “some concerns” for risk of bias, and the remaining twelve studies had “high concerns” for risk of bias. Concerns surrounding the risk of bias predominantly surrounded lack of clarity regarding the randomization process, and due to universal prevention studies utilizing cluster randomization. The weighted risk of bias is presented as a plot in Figure 3.

#### Effect size analysis

The meta-analyses were completed to compare the intervention and control of the primary outcomes (internalizing problems) at post-intervention and 6- and 12-month follow-up. MAJOR was used to conduct the meta-analysis, and a random-effects model was used to weight the primary studies. Results for each outcome variable are provided within the Supplementary material.

#### Measures of internalizing symptoms

**Figure 4:**
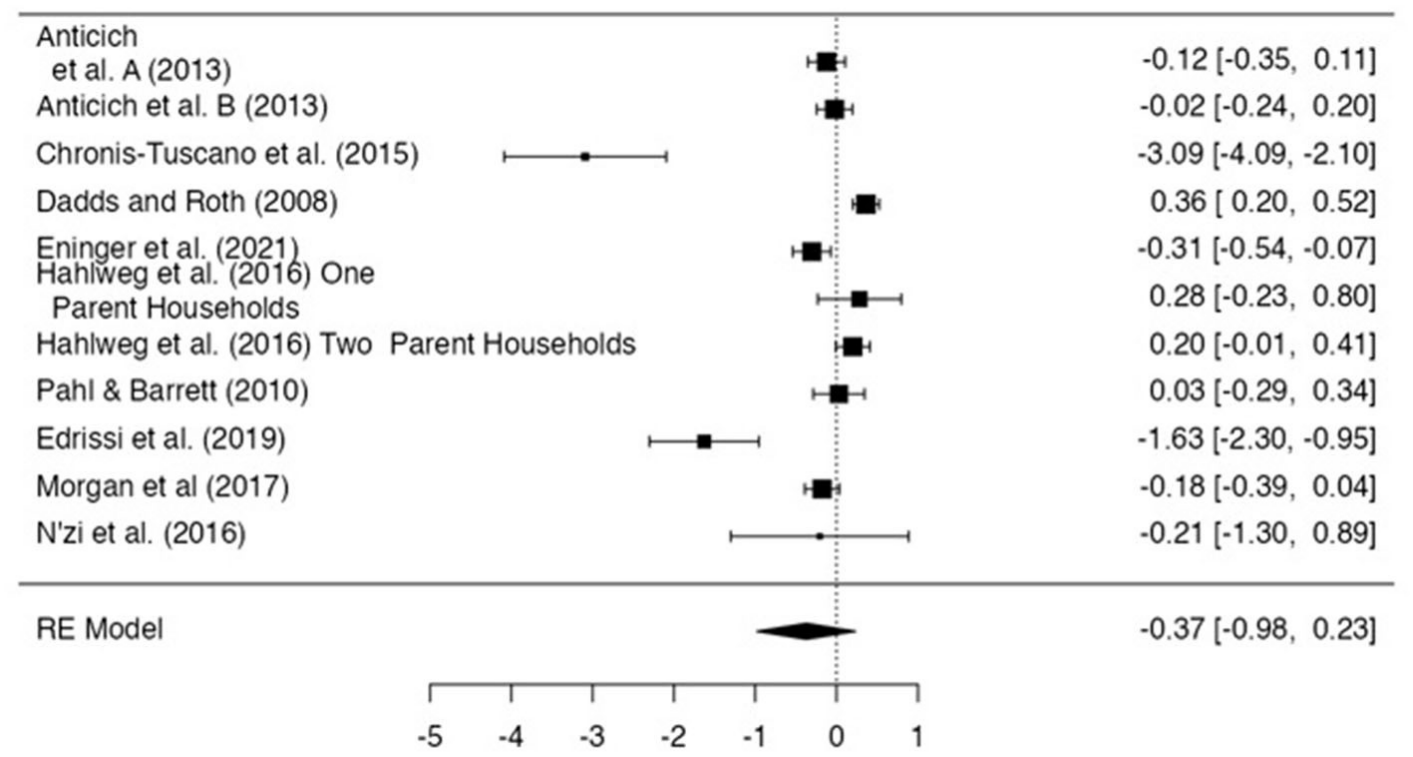
Effect size for internalizing problem score. Anticich et al. (2013) denoted the intervention group and Anticich et al. (2013) denotes the active control group.

The overall effect size at post-intervention for the prevention of internalizing symptoms estimated a non-significant small-to-moderate effect size of−0.37 (*n* = 11, *p* = 0.23 CI −0.98, 0.23; Figure 4). There was significant heterogeneity (*I*^2^ = 98.1) found. Moderators (targeted/universal, caregiver intervention/child intervention) were entered to attempt to explain this heterogeneity, however, no significant relationships were found (see Table 2).

**Table 2:** Meta-analysis and supplementary analyses for internalizing symptoms: analysis of standardized difference in means (hedges g).

**Analysis and variable**	**Number of comparisons (k)**	**Estimate (SE)**	**95% CI**	***z***	***p***	***Q*-value**	***I^2^***
Internalizing symptoms (pre-post)	11	−0.37(0.31)	CI: −0.98 to 0.23	−1.20	0.23	93.54	98.08%
Follow-up: 6- to 7-months	7	−0.38 (0.16)	CI: −0.69 to −0.06	−2.35	0.02^*^	33.19	82.99%
Follow-up: 12-months	6	−0.03(0.15)	−0.32 to 0.25	−0.24	0.81	19.51	84.78%
**Subgroup/moderator analysis**							
Universal intervention (pre-post)	7	0.05 (0.08)	−0.12 to 0.22	0.59	0.54	27.73	70.11
Targeted intervention (pre-post)	4	−1.25 (0.69)	−0.26 to 0.1	−1.81	0.07	45.09	94.17
Intervention provided to children (pre-post)	3	−0.15 (0.08)	0.31 to 0.01	−1.8	0.07	2.99	33.33%
Intervention provided to parent/caregiver(s) (pre-post)	6	−0.15 (0.31)	−0.73 to −0.43	−0.51	0.61	43.03	95.63%

* refers to the result being significant at the *p* < 0.05 level.

#### Publication bias

There was some evidence of publication bias, as evidenced by inspection of the funnel plot. Additionally, Egger et al. (1997) regression test also showed funnel plot asymmetry (*z* = −2.37, *p* = 0.02). See Supplementary material for the funnel plot.

#### Six-and seven-month follow-up

**Figure 5:**
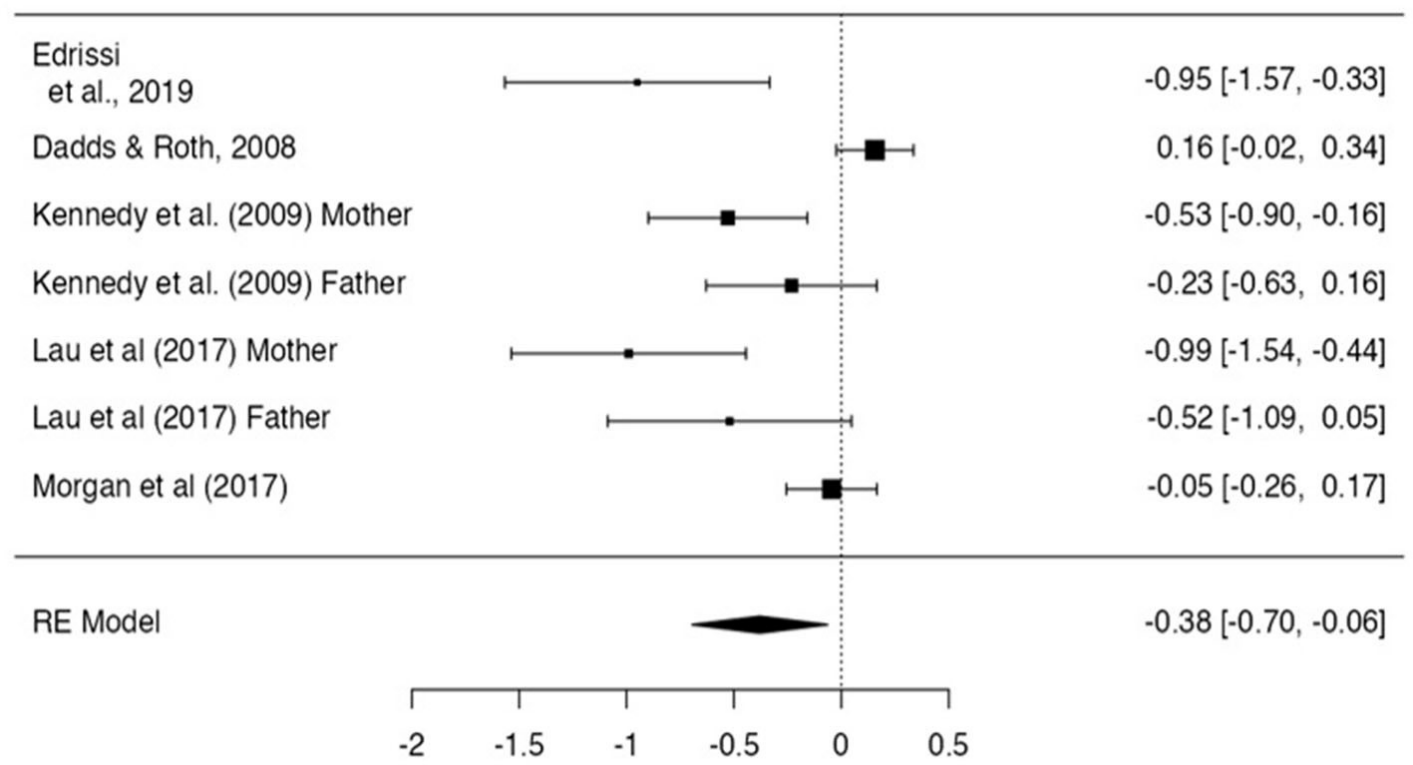
Effect size for internalizing problem scores- 6 and 7 month follow up.

The overall effect size at 6- and 7-month post-intervention estimated a significant small-to-moderate effect size of −0.38 (*n*= 7, *p* = 0.02, CI −0.69, −0.06; Figure 5). Egger's regression test indicated some evidence of publication bias (*z* = −2.35, *p* = 0.02) and significant heterogeneity (*I*^2^ = 83) was found.

#### 12-month follow-up

**Figure 6:**
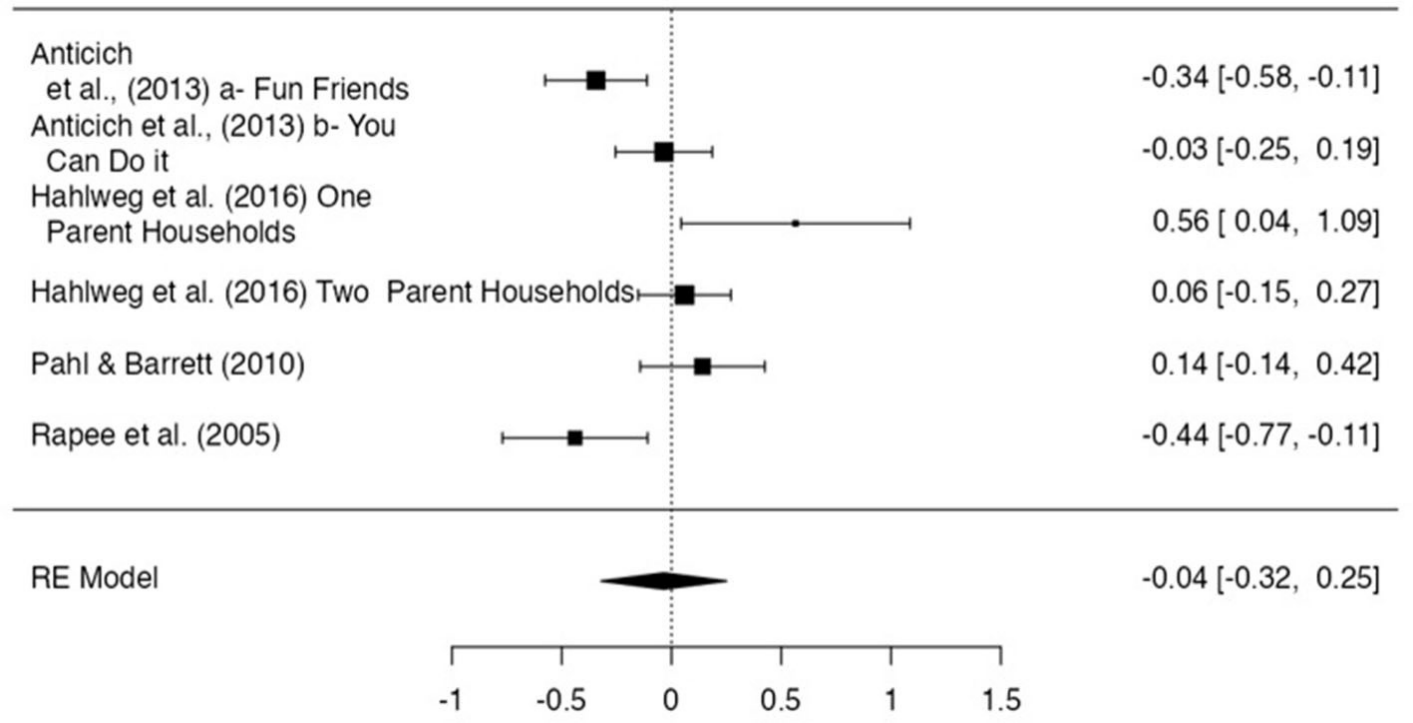
Effect size for internalizing problem scores−12 month follow-up.

The overall effect size at 6-and 7-month post-intervention estimated a non-significant negligible effect size of −0.03 (*n* = 6, *p* = 0.81, CI −0.32, 0.25; Figure 6). Egger's regression test did not indicate evidence of publication bias (z- 1.19, *p* = 0.23). Significant heterogeneity (*I*^2^ = 84.78) was found.

## Discussion

This is the first meta-analysis specifically focusing on children aged 3- to 5-years, that reviewed the effects of both universal, and targeted interventions for both parents and children. The aim of the systematic review and meta-analysis was to determine whether social and emotional programs were effective for the prevention of internalizing disorders in early childhood (3- to 5-years), and in which characteristics of the featured programs were related to the efficacy of the programs in reducing internalizing disorders. Overall, the study included 3,381 children and identified 17 studies, consisting of seven universal prevention programs, and ten targeted prevention programs. Results of the meta-analyses revealed that prevention programs contributed a small-to-moderate significant effect size in reducing internalizing problems at 6-and 7-month post-intervention. However, no significant effect was found post-intervention, or in the 12-month follow-up period. There was significant heterogeneity in effect sizes between studies in all meta-analyses, however, neither the type of the prevention (i.e., universal, or targeted) nor whom the prevention was provided to (i.e., caregiver[s] or children) were found to be significant moderators of variance. While the 6- and 7-month post-intervention showed a significant effect, there is varying evidence to conclude that the universal and targeted programs are effective for the prevention of internalizing disorders. No included studies were rated as being low in bias, and there was significant funnel plot asymmetry indicating publication bias.

In synthesizing the results reported in the current study, it appears that social and emotional programs have a small-to-moderate effect on internalizing problems in the 6 months following the programs. This is consistent with other meta-analyses that have reported that studies often only detect significant changes when long-term follow-up assessments have been completed (Stockings et al., 2016; Werner-Seidler et al., 2017). Caldwell et al. (2019) discussed a trend where previous meta-analyses generally have shown small but significant prevention effects at 6-to-12 months, with effects tending to deteriorate by 12-month follow-up. It has been proposed that prevention programs may need a 6-to-12-month follow-up before significant change is identified, as prevention effects may only emerge when children experience increased risk, which may only emerge over time. It is also important to acknowledge that the results reported from the period immediately after the intervention, 6- to 7-month and 12-month follow-up may also not necessarily be from the same studies, as several studies only reported at a particular time point (e.g., only at 6-month follow up). This sampling difference may have also contributed to the differences in effects between time points, particularly at 6- and 12-month follow-up. Additionally, other meta-analyses (Fisak, 2014; Stockings et al., 2016; Werner-Seidler et al., 2017) addressing prevention often include data from children and adolescents and do not assess at a particular age period, as our analyses have done. Caldwell et al. (2019) proposed that direct comparisons including a wide range of age groups may not be appropriate, as young children generally exhibit less anxiety compared to adolescents. The differences in symptoms in an early childhood sample may be too small to find a prevention effect as there is less opportunity for improvement in scores.

The current study also reviewed characteristics of studies and how they related to the effectiveness of the programs in reducing internalizing problems. This was important in informing future directions and planning of social and emotional prevention programs. There appeared to be no significant effect when addressing whether programs were delivered to children or parents. None of the tested characteristics seemed to stand out as being associated with bigger effects.

Targeted programs that focused on inhibited and anxious children seemed to contribute to decreased internalizing symptoms more than universal interventions, although still not significantly. This was congruent with other meta-analyses that have previously assessed universal prevention programs which have also reported negligible-to-small effect sizes on internalizing problems (Yap et al., 2016; Werner-Seidler et al., 2017; Caldwell et al., 2019). Our results were also consistent with (Werner-Seidler et al., 2017) meta-analyses that reviewed 81 RCTs, where they only found a small effect on depression and anxiety and found that targeted programs showed greater effect sizes as opposed to universal programs. Universal programs may appear to be less effective than targeted programs as they are not directed at sub-clinical populations (i.e., inhibited children). It is thought that universal prevention programs aimed at broader and low-risk populations have less breadth for improvement, and thus results may be due to a floor effect. While targeted prevention programs may have more significant effects as they are more focused on children who are at greater risk of developing a disorder. While universal prevention effects appear to be less likely to be found as they include the broader population who are not at risk. However, due to universal programs being designed to target a broader population, even small effects of universal programs that are clinically significant are influential (Werner-Seidler et al., 2017). Compared to targeted programs, universal programs can intervene at a macro level, are more cost effective, may support children at risk of being overlooked, promote mental health literacy, and reduce stigmatization (Baughman et al., 2020).

## Limitations and future research directions

Our results should be interpreted in the context of some limitations, including the identification and extraction of studies. The study searched three electronic databases, with seventeen research studies identified. Previous meta-analyses have been far broader and assessed a far greater number of studies due to having fewer constraints in their exclusion criteria. While this may be interpreted as a benefit, as to our knowledge, no previous meta-analyses have specifically assessed the early childhood period, it also limits the breadth of our findings. While including only RCTs was considered more rigorous and thought to contribute to the quality of studies included, it also limited the number of studies being reviewed. Future studies may broaden the inclusion criteria by including a wider range of research designs, for example, quasi-experimental and pre-post (e.g., Oorloff et al., 2021). Including a wider range of research designs may support the further investigation of new and emerging prevention programs. Furthermore, the search was limited by language bias, with the language barrier of the reviewers limiting the study, resulting in the selection of English-only papers. Moreover, while authors of the included studies were contacted to obtain missing data, authors were only provided with a brief period to respond due to time constraints surrounding the data analysis meaning that five otherwise eligible studies were excluded (see Supplementary material). It is possible that the inclusion of these studies might have modified the validity and conclusion of the review.

Additionally, a significant limitation was the evidence of risk of bias. No studies were rated as “low risk of bias”, and the meta-analyses exhibited evidence of publication bias, which may have inflated effect sizes. The “file draw problem”, where studies with statistically significant results are more likely to be published than those findings that have no significant differences (Wagner III, 2022) is important to be acknowledged. It is unknown how many unpublished studies with non-significant results were unavailable on these sources. Publication bias creates confirmation bias toward successfully published literature, which can affect the accurate representation of the true intervention effect. However, some studies utilized the publication of study protocols, and the increasing trend of this practice should reduce the selective publication of studies reporting group differences. Future reviewers may consider broadening their inclusion criteria and literature to maximize the breadth of their findings and to reduce potential publication bias.

An additional risk of bias included a lack of transparency surrounding the randomization process and the use of cluster randomization. The quality of prevention programs may be improved through using random-sequencing methods and using adequate allocation concealment, which few studies reported using. While it is plausible that many studies followed these methods, most studies did not provide sufficient information surrounding possible selection bias. All studies utilizing universal prevention programs used cluster randomization, given they all took place within school settings. Most studies utilizing cluster randomization were flagged in the randomization process during the RoB2 assessment and were rated as “high risk” for risk of bias. The main concern surrounding cluster designs is that participants in a cluster (i.e., a classroom or primary school as a whole) may respond similarly, and the results cannot be assumed to be independent (Eninger et al., 2021).

Future research would benefit from identifying the characteristics which improve program retention and follow up outcomes (Hahlweg et al., 2010). This study sought to identify differences in outcomes for programs delivered to parents and young children, along with targeted and universal programs. Additional research which considers specific factors of intervention, such as coping skills being developed (Bernaras et al., 2019). Furthermore, our study is limited by focusing on prevention of symptoms and future reviews may benefit from inclusion of interventions targeted toward reducing symptoms in young children already presenting with internalizing problems (Blewitt et al., 2021).

## Conclusion

Findings from the current systematic review and meta-analysis suggest that there may be value in ongoing development and evaluation of prevention programs for internalizing programs for children aged 3-to-5 years, as they impact students and reduce internalizing difficulties within the 6- to 7- month timeframe following prevention programs. However, results should be interpreted with the provision that the overall quality of the included studies was low, and there was significant heterogeneity. Future individual studies should prioritize minimizing the use of clustered randomization, publish study protocols, and report clearer method and randomization approaches. Future meta-analyses should also involve the incorporation of a wider range of inclusion criteria (e.g., increased research designs) and should also involve grouping research by age range (e.g., preschool, middle years, and youth) to determine if combining of age groups is supporting a floor effect.

Due to mental health difficulties in early life having a significant impact on future health, it is vital to build the foundations of social and emotional learning during early development. Prevention programs are a proactive and macro approach to preventing psychopathology and support children's development of social-emotional well-being. Given the significant impact of internalizing problems for developing children, it is worth continuing to pursue the development and evaluation of prevention programs, as these support children and communities to flourish.

## Data availability statement

The original contributions presented in the study are included in the article/Supplementary material, further inquiries can be directed to the corresponding author.

## Author contributions

All authors listed have made a substantial, direct, and intellectual contribution to the work and approved it for publication.
