# Supplementary material for: Systematic review and meta-analysis of the prevention of internalizing disorders in early childhood
Source: Front Psychol. 2023 Dec 14;14:1061825. doi: 10.3389/fpsyg.2023.1061825 (PMC10752964; doi:10.3389/fpsyg.2023.1061825)
Supplement: Supplementary file 1 [file Data_Sheet_1.PDF]

Supplementary Material

Supplementary Material A

Search terms for each database

#	PsycINFO
1	major depression/ or dysthymic disorder/ or atypical depression/ or internalizing symptoms/
2	anxiety disorders/ or generalized anxiety disorder/ or obsessive compulsive disorder/ or panic attack/ or panic disorder/ or phobias/ or separation anxiety disorder/ or trichotillomania/ or anxiety/ or anxiety management/ or social anxiety/ or speech anxiety/
3	internalizing symptoms/
4	internalising symptoms/ or internalising problems/ or internalising/ or internalisation.mp. [mp=title, abstract, heading word, table of contents, key concepts, original title, tests & measures, mesh]
5	school-based intervention/ or early intervention/ or children/ or parenting/ or parent training/ or parenting intervention/ or family intervention.mp. [mp=title, abstract, heading word, table of contents, key concepts, original title, tests & measures, mesh]
6	prevent/ or prevention/ or school prevention/ or intervention/ or treatment/ or family intervention/ or early intervention/ or child/ or youth/ or children/ or preschool children/ or preschool.mp. [mp=title, abstract, heading word, table of contents, key concepts, original title, tests & measures, mesh]
7	1 or 2 or 3 or 4
8	5 or 6
9	7 and 8
10	limit 9 to 100 childhood

#	Embase
**1**	(Internal*ng or Internali*ation).mp. [mp=title, abstract, heading word, drug trade name, original title, device manufacturer, drug manufacturer, device trade name, keyword, floating subheading word, candidate term word]
**2**	Depression/ or depressive disorder, major/ or anxiety/ or anxiety, separation/ or anxiety disorders/ or performance anxiety/ or agoraphobia/ or obsessive-comulsive disorder/ or panic disorder/ or phobic disorders/ or phobia, social.mp. [mp=title, abstract, heading word, drug trade name, original title, device manufacturer, drug manufacturer, device trade name, keyword, floating subheading word, candidate term word]

**3**	exp behavior disorder/
**4**	early intervention/ or school based intervention/ or parenting intervention/ or parent training/ or family intervention.mp. [mp=title, abstract, heading word, drug trade name, original title, device manufacturer, drug manufacturer, device trade name, keyword, floating subheading word, candidate term word]
**5**	child, preschool/ or child.mp. [mp=title, abstract, heading word, drug trade name, original title, device manufacturer, drug manufacturer, device trade name, keyword, floating subheading word, candidate term word]
**6**	1 or 2 or 3
**7**	4 and 5 OR 6

#	MEDLINE
1	depression/ or depressive disorder/ or major depression/ or dysthymic disorder.mp. [mp=title, abstract, original title, name of substance word, subject heading word, floating sub-heading word, keyword heading word, organism supplementary concept word, protocol supplementary concept word, rare disease supplementary concept word, unique identifier, synonyms]
2	anxiety/ or generalised anxiety/ or panic disorder/ or social phobia/ or agoraphobia/ or obsessive compulsive disorder/ or anxiety disorders.mp. [mp=title, abstract, original title, name of substance word, subject heading word, floating sub-heading word, keyword heading word, organism supplementary concept word, protocol supplementary concept word, rare disease supplementary concept word, unique identifier, synonyms]
3	child behaviour/ or internalising/ or internalisation.mp. [mp=title, abstract, original title, name of substance word, subject heading word, floating sub-heading word, keyword heading word, organism supplementary concept word, protocol supplementary concept word, rare disease supplementary concept word, unique identifier, synonyms]
4	school-based intervention/ or early intervention/ or parenting/ or children/ or parenting training/ or parenting intervention/ or family intervention/ or youth/ or preschool children.mp. [mp=title, abstract, original title, name of substance word, subject heading word, floating sub-heading word, keyword heading word, organism supplementary concept word, protocol supplementary concept word, rare disease supplementary concept word, unique identifier, synonyms]
5	1 or 2
6	3 or 4
7	5 and 6
8	limit 7 to "preschool child (2 to 5 years)"

Supplementary Material B

Extracted Data

	Intervention						Control						Intervention			Control		
	Pre			Post			Pre			Post			Follow Up			Follow Up		
**Study**	*N*	*M*	*SD*	*N*	*M*	*SD*	*N*	*M*	*SD*	*N*	*M*	*SD*	*N*	*M*	*SD*	*N*	*M*	*SD*
Anticich et al., (2013) a- Fun Friends	159	24.85	10.54	159	19.67	11.47	133	23.52	12.03	133	21.16	12.54	159	16.39	9.92	133	20.05	11.4
Anticich et al., (2013) b- You Can Do it	196	24.61	11.53	196	20.85	12.6	133	23.52	12.03	133	21.16	12.54	196	19.64	12	133	20.05	11.4
Chronis-Tuscano et al. (2015)	18	60.83	1.19	17	51.02	2.19	22	58.72	2.1	17	58.05	2.25						
Dadds & Roth, 2008	336	39.45	7.8	272	41.68	7.33	373	38.98	8.36	321	38.79	8.49	228	41.72	7.67	252	40.51	7.8
Edrissi et al., 2019	23	41.91	7.13	23	32.65	6.01	22	45.82	6.93	22	43.68	7.27	23	34.87	8.17	22	42.36	7.27
Eninger et al. (2021)	145	0.37	0.517	145	0.397	0.546	140	0.549	0.581	140	0.577	0.627						
Hahlweg et al. (2016) One Parent Households	28	10.8	8.6	28	10.1	8.5	31	10	8.5	31	8.1	5.3	28	12.1	10.6	31	7.3	5.7
Hahlweg et al. (2016) Two Parent Households	296	8.6	6	296	6.9	5.8	122	7.3	5.3	122	5.8	4.7	296	5.9	5.3	122	5.6	4.4
Kennedy et al. (2009) Mother	58	63.16	16.11				58	63.7	16.6				58	50.6	18.39	58	61.04	20.79

Kennedy et al. (2009) Father	49	51.76	20.48				49	57.26	20.57				49	49.82	14.15	49	52.88	11.88
Lau et al (2017) Mother	39	63.15	16.11				31	63.7	16.6				36	44.74	16.74	24	63.28	20.85
Lau et al (2017) Father	37	51.76	20.48				30	57.26	20.57				32	46.38	20.36	20	59.9	32.38
Morgan et al (2017)	215	5.6	2.3	156	4.1	2.3	218	5.5	2.2	187	4.5	2.2	166	4	2.2	180	4.1	2.2
N'zi et al. (2016)	7	59.43	12.79	7	53.29	6.78	5	63.29	8.76	6	55.57	13.29						
Pahl & Barrett (2010)	134	21.88	11.55	79	18.56	10.67	129	19.85	11.47	76	18.24	11.2						
Rapee et al. (2005)	73	2.1	1.2				72	1.8	1.1				72	0.7	0.8	72	1.1	1

Supplementary Material C

Missing Data- Emailed Researchers

	Reason Why	Intervention						Control						Intervention			Control		
		Pre			Post			Pre			Post			Follow Up			Follow Up		
**Study**		N	M	SD	N	M	SD	N	M	SD	N	M	SD	N	M	SD	N	M	SD
Domitriovich et al., 2007 a - Parents Report	No participant numbers per group provided		0.81	0.42	201	0.87	0.4		0.85	0.4		0.96	0.45						
Domitriovich et al., 2007 b- Teachers Report	No participant numbers per group provided		0.58	0.6	201	0.61	0.58		0.87	0.47		0.85	0.56						
Fishbein et al. (2016)	Only model adjusted means estimates																		
Cartwright-Hatton et al. (2018)	No raw data provided														2.5	2		2.9	2.2
Carta et al. (2013)	No baseline/pre scores provided														48.48	1.84		49.21	10.35
Bayer et al. (2018)	No baseline/pre scores provided																		

Supplementary Material D

PRISMA Checklist

Section and Topic	Item #	Checklist item	Location where item is reported
**TITLE**			
Title	1	Identify the report as a systematic review.	i
**ABSTRACT**			
Abstract	2	See the PRISMA 2020 for Abstracts checklist.	1
**INTRODUCTION**			
Rationale	3	Describe the rationale for the review in the context of existing knowledge.	6
Objectives	4	Provide an explicit statement of the objective(s) or question(s) the review addresses.	6
**METHODS**			
Eligibility criteria	5	Specify the inclusion and exclusion criteria for the review and how studies were grouped for the syntheses.	7
Information sources	6	Specify all databases, registers, websites, organisations, reference lists and other sources searched or consulted to identify studies. Specify the date when each source was last searched or consulted.	9
Search strategy	7	Present the full search strategies for all databases, registers and websites, including any filters and limits used.	7,8,9
Selection process	8	Specify the methods used to decide whether a study met the inclusion criteria of the review, including how many reviewers screened each record and each report retrieved, whether they worked independently, and if applicable, details of automation tools used in the process.	9
Data collection process	9	Specify the methods used to collect data from reports, including how many reviewers collected data from each report, whether they worked independently, any processes for obtaining or confirming data from study investigators, and if applicable, details of automation tools used in the process.	9
Data items	10a	List and define all outcomes for which data were sought. Specify whether all results that were compatible with each outcome domain in each study were sought (e.g. for all measures, time points, analyses), and if not, the methods used to decide which results to collect.	10,11
	10b	List and define all other variables for which data were sought (e.g. participant and intervention characteristics, funding sources). Describe any assumptions made about any missing or unclear information.	10,11
Study risk of bias assessment	11	Specify the methods used to assess risk of bias in the included studies, including details of the tool(s) used, how many reviewers assessed each study and whether they worked independently, and if applicable, details of automation tools used in the process.	13
Effect measures	12	Specify for each outcome the effect measure(s) (e.g. risk ratio, mean difference) used in the synthesis or presentation of results.	14,15

Section and Topic	Item #	Checklist item	Location where item is reported
Synthesis methods	13a	Describe the processes used to decide which studies were eligible for each synthesis (e.g. tabulating the study intervention characteristics and comparing against the planned groups for each synthesis (item #5)).	14,15
	13b	Describe any methods required to prepare the data for presentation or synthesis, such as handling of missing summary statistics, or data conversions.	14,15
	13c	Describe any methods used to tabulate or visually display results of individual studies and syntheses.	14,15
	13d	Describe any methods used to synthesize results and provide a rationale for the choice(s). If meta-analysis was performed, describe the model(s), method(s) to identify the presence and extent of statistical heterogeneity, and software package(s) used.	14,15
	13e	Describe any methods used to explore possible causes of heterogeneity among study results (e.g. subgroup analysis, meta-regression).	14,15
	13f	Describe any sensitivity analyses conducted to assess robustness of the synthesized results.	14,15
Reporting bias assessment	14	Describe any methods used to assess risk of bias due to missing results in a synthesis (arising from reporting biases).	14,15
Certainty assessment	15	Describe any methods used to assess certainty (or confidence) in the body of evidence for an outcome.	14,15
**RESULTS**			
Study selection	16a	Describe the results of the search and selection process, from the number of records identified in the search to the number of studies included in the review, ideally using a flow diagram.	10, 16
	16b	Cite studies that might appear to meet the inclusion criteria, but which were excluded, and explain why they were excluded.	N/A
Study characteristics	17	Cite each included study and present its characteristics.	12
Risk of bias in studies	18	Present assessments of risk of bias for each included study.	13, 14
Results of individual studies	19	For all outcomes, present, for each study: (a) summary statistics for each group (where appropriate) and (b) an effect estimate and its precision (e.g. confidence/credible interval), ideally using structured tables or plots.	20,21
Results of syntheses	20a	For each synthesis, briefly summarise the characteristics and risk of bias among contributing studies.	20,21
	20b	Present results of all statistical syntheses conducted. If meta-analysis was done, present for each the summary estimate and its precision (e.g. confidence/credible interval) and measures of statistical heterogeneity. If comparing groups, describe the direction of the effect.	20, 21, 22
	20c	Present results of all investigations of possible causes of heterogeneity among study results.	20, 21, 22

Section and Topic	Item #	Checklist item	Location where item is reported
	20d	Present results of all sensitivity analyses conducted to assess the robustness of the synthesized results.	20, 21, 22
Reporting biases	21	Present assessments of risk of bias due to missing results (arising from reporting biases) for each synthesis assessed.	20, 21, 22
Certainty of evidence	22	Present assessments of certainty (or confidence) in the body of evidence for each outcome assessed.	20, 21, 22
**DISCUSSION**			
Discussion	23a	Provide a general interpretation of the results in the context of other evidence.	
	23b	Discuss any limitations of the evidence included in the review.	
	23c	Discuss any limitations of the review processes used.	
	23d	Discuss implications of the results for practice, policy, and future research.	
**OTHER INFORMATION**			
Registration and protocol	24a	Provide registration information for the review, including register name and registration number, or state that the review was not registered.	7
	24b	Indicate where the review protocol can be accessed, or state that a protocol was not prepared.	7
	24c	Describe and explain any amendments to information provided at registration or in the protocol.	7
Support	25	Describe sources of financial or non-financial support for the review, and the role of the funders or sponsors in the review.	N/A
Competing interests	26	Declare any competing interests of review authors.	N/A
Availability of data, code and other materials	27	Report which of the following are publicly available and where they can be found: template data collection forms; data extracted from included studies; data used for all analyses; analytic code; any other materials used in the review.	N/A

Supplementary Material E. Measures of internalizing symptoms

1 Mean Differences (n, M, SD)

Random-Effects Model (k = 11)

	Estimate	se	Z	p	CI Lower Bound	CI Upper Bound
Intercept	-0.373	0.310	-1.20	0.229	-0.980	0.234
	.	.	.	.	.	.

Note. Tau^2 Estimator: Hedges

Heterogeneity Statistics

Tau	Tau^2	I^2	H^2	R^2	df	Q	p
0.994	0.9871 (SE= 0.4782)	98.08%	52.093	.	10.000	93.539	< .001

1.1 Forest Plot

Publication Bias Assessment

Test Name	value	p
Fail-Safe N	21.000	0.003
Begg and Mazumdar Rank Correlation	-0.418	0.087
Egger's Regression	-2.371	0.018
Trim and Fill Number of Studies	0.000	.

Note. Fail-safe N Calculation Using the Rosenthal Approach

Supplementary Material F. Six and Seven Month Follow up

2 Mean Differences (n, M, SD)

Random-Effects Model (k = 7)

	Estimate	se	Z	p	CI Lower Bound	CI Upper Bound
Intercept	-0.380	0.162	-2.35	0.019	-0.696	-0.063
	.	.	.	.	.	.

Note. Tau² Estimator: Hedges

Heterogeneity Statistics

Tau	Tau²	I^2	H^2	R^2	df	Q	p
0.371	0.1375 (SE= 0.1103)	82.99%	5.878	.	6.000	33.190	< .001

Publication Bias Assessment

Test Name	value	p
Fail-Safe N	38.000	< .001
Begg and Mazumdar Rank Correlation	-0.619	0.069
Egger's Regression	-5.384	< .001

Publication Bias Assessment

Test Name	value	p
Trim and Fill Number of Studies	3.000	.

Note. Fail-safe N Calculation Using the Rosenthal Approach

2.1 Funnel Plot

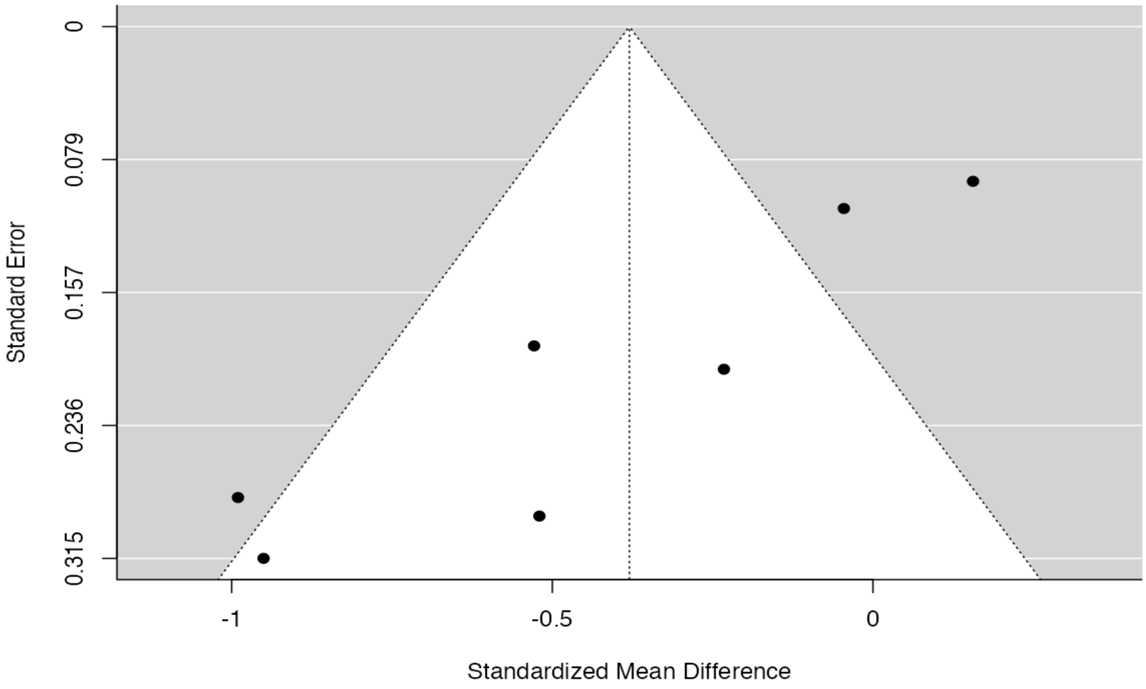

Supplementary Material G. 12-month follow up

3 Mean Differences (n, M, SD)

Random-Effects Model (k = 6)

	Estimate	se	Z	p	CI Lower Bound	CI Upper Bound
Intercept	-0.0351	0.146	-0.240	0.810	-0.322	0.251
	.	.	.	.	.	.

Note. Tau² Estimator: Hedges

Heterogeneity Statistics

Tau	Tau²	I²	H²	R²	df	Q	p
0.323	0.1045 (SE= 0.0836)	84.78%	6.569	.	5.000	19.513	0.002

Publication Bias Assessment

Test Name	value	p
Fail-Safe N	0.000	0.188
Begg and Mazumdar Rank Correlation	0.200	0.719
Egger's Regression	1.186	0.236
Trim and Fill Number of Studies	1.000	.

Note. Fail-safe N Calculation Using the Rosenthal Approach

3.1 Funnel Plot

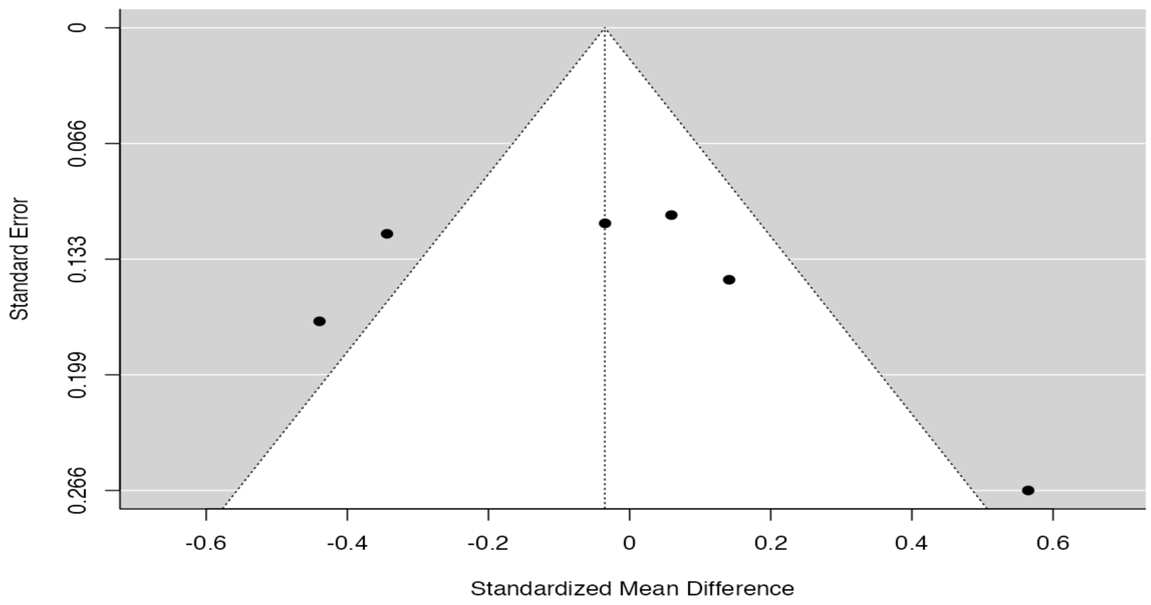

Supplementary Material H: Subgroup/Moderator Analysis – Universal Intervention

Meta-Analysis

Random-Effects Model (k = 7)

	Estimate	se	Z	p	CI Lower Bound	CI Upper Bound
Intercept	0.0514	0.0858	0.599	0.549	-0.117	0.219
	.	.	.	.	.	.

Note. Tau^2 Estimator: Hedges

Heterogeneity Statistics

Tau	Tau²	I²	H²	R²	df	Q	p
0.184	0.034 (SE= 0.0339)	70.11%	3.345	.	6.000	27.773	< .001

Forest Plot

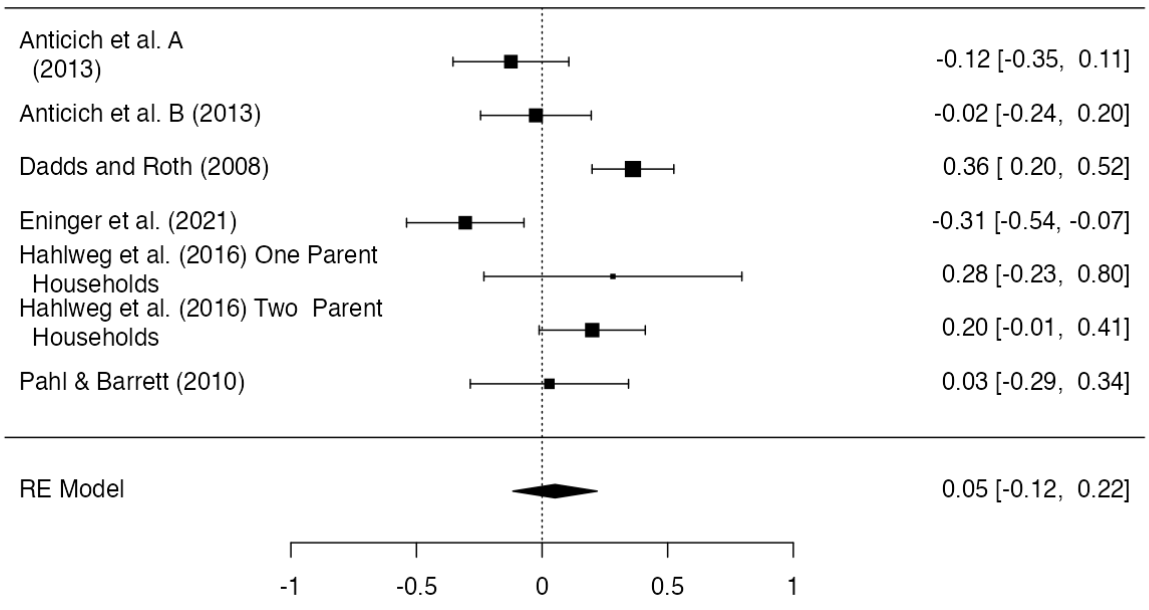

Publication Bias Assessment

Test Name	value	p
Fail-Safe N	0.000	0.085
Begg and Mazumdar Rank Correlation	-0.333	0.381
Egger's Regression	0.108	0.914
Trim and Fill Number of Studies	0.000	.

Note. Fail-safe N Calculation Using the Rosenthal Approach

Funnel Plot

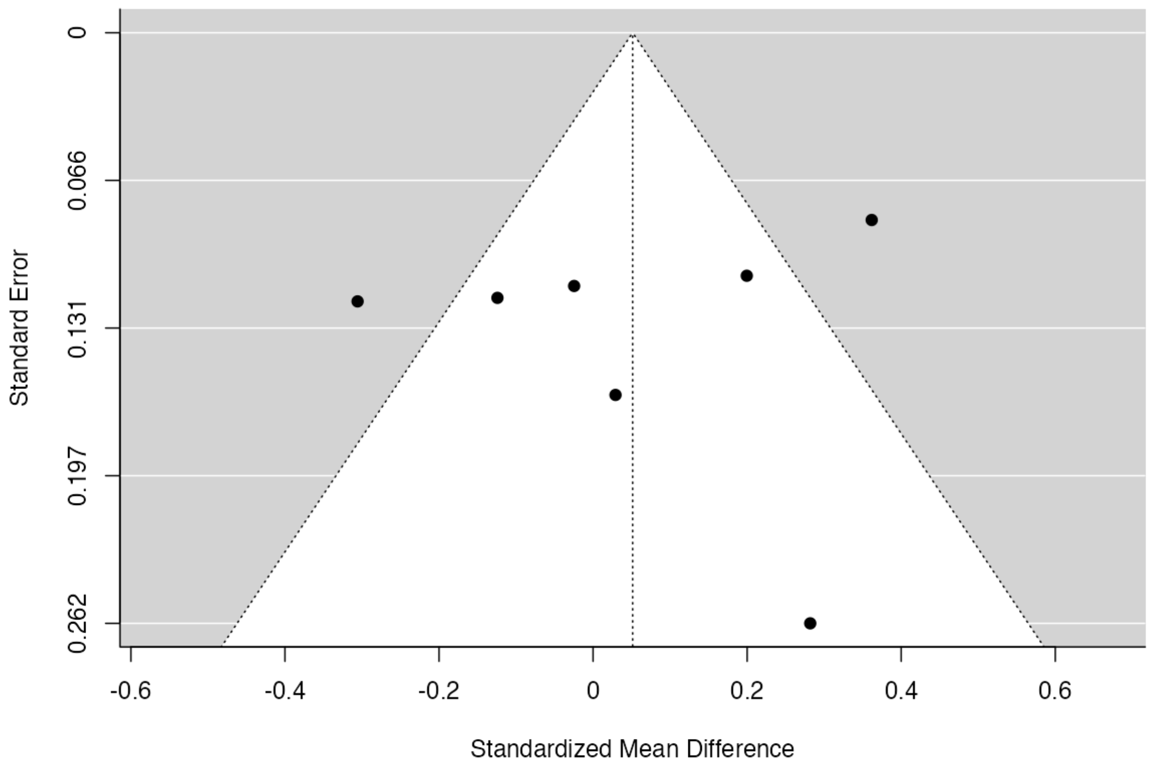

Supplementary Material I. Subgroup/Moderator Analysis – Targeted Intervention

Mean Differences (n, M, SD)

Random-Effects Model (k = 4)

	Estimate	se	Z	p	CI Lower Bound	CI Upper Bound
Intercept	-1.25	0.692	-1.81	0.070	-2.609	0.104

Random-Effects Model (k = 4)

	Estimate	se	Z	p	CI Lower Bound	CI Upper Bound
	.	.	.	.	.	.

Note. Tau^2 Estimator: Hedges

Heterogeneity Statistics

Tau	Tau^2	I^2	H^2	R^2	df	Q	p
1.322	1.7484 (SE= 1.5723)	94.17%	17.141	.	3.000	45.088	< .001

Forest Plot

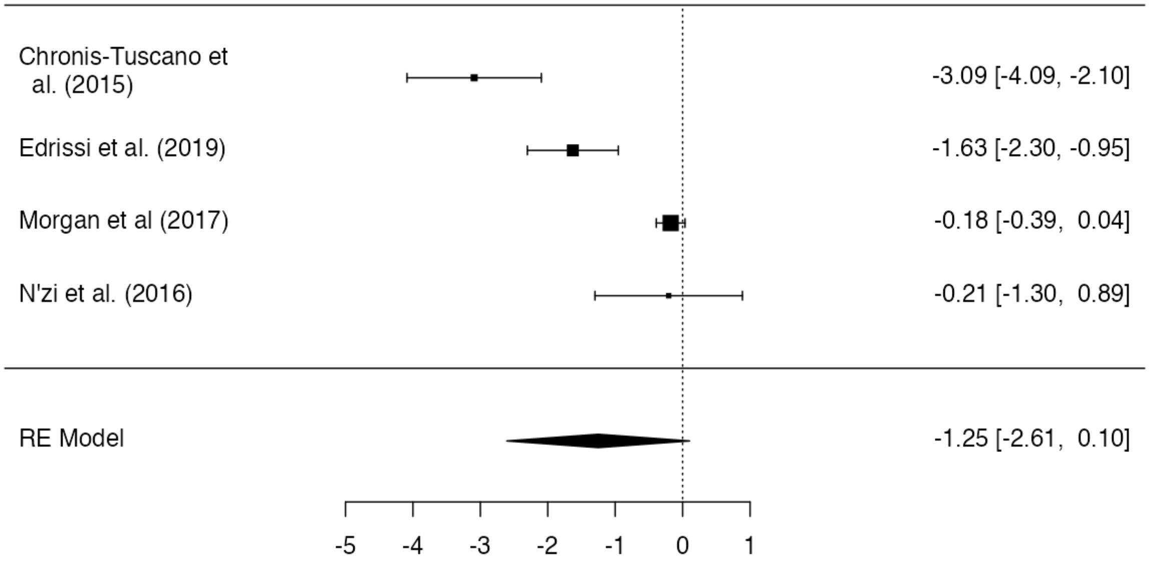

Publication Bias Assessment

Test Name	value	p
Fail-Safe N	57.000	< .001
Begg and Mazumdar Rank Correlation	-0.333	0.750
Egger's Regression	-0.636	0.525
Trim and Fill Number of Studies	0.000	.

Note. Fail-safe N Calculation Using the Rosenthal Approach

Funnel Plot

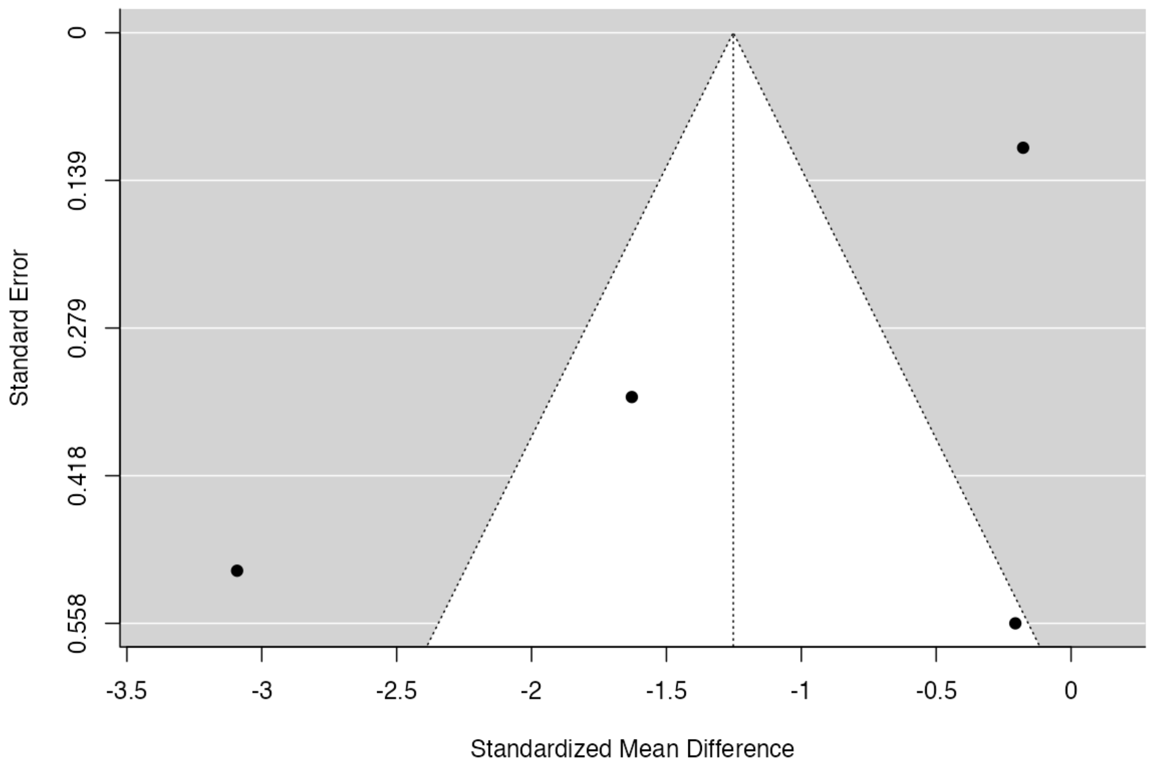

Supplementary Material H. **Subgroup/Moderator Analysis – Intervention provided to children**

Meta-Analysis

Random-Effects Model (k = 3)

	Estimate	se	Z	p	CI Lower Bound	CI Upper Bound
Intercept	-0.148	0.0823	-1.80	0.072	-0.309	0.013
	.	.	.	.	.	.

Note. Tau² Estimator: Hedges

Heterogeneity Statistics

Tau	Tau²	I²	H²	R²	df	Q	p
0.082	0.0068 (SE= 0.0203)	33.33%	1.500	.	2.000	2.999	0.223

Forest Plot

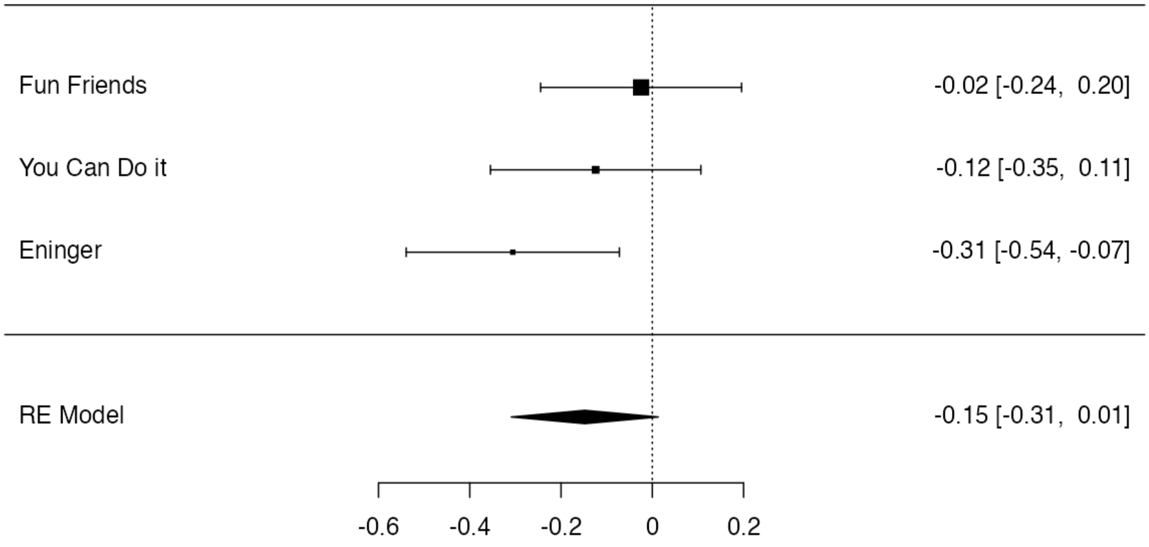

Publication Bias Assessment

Test Name	value	p
Fail-Safe N	3.000	0.013
Begg and Mazumdar Rank Correlation	-1.000	0.333
Egger's Regression	-1.548	0.122
Trim and Fill Number of Studies	0.000	.

Note. Fail-safe N Calculation Using the Rosenthal Approach

Funnel Plot

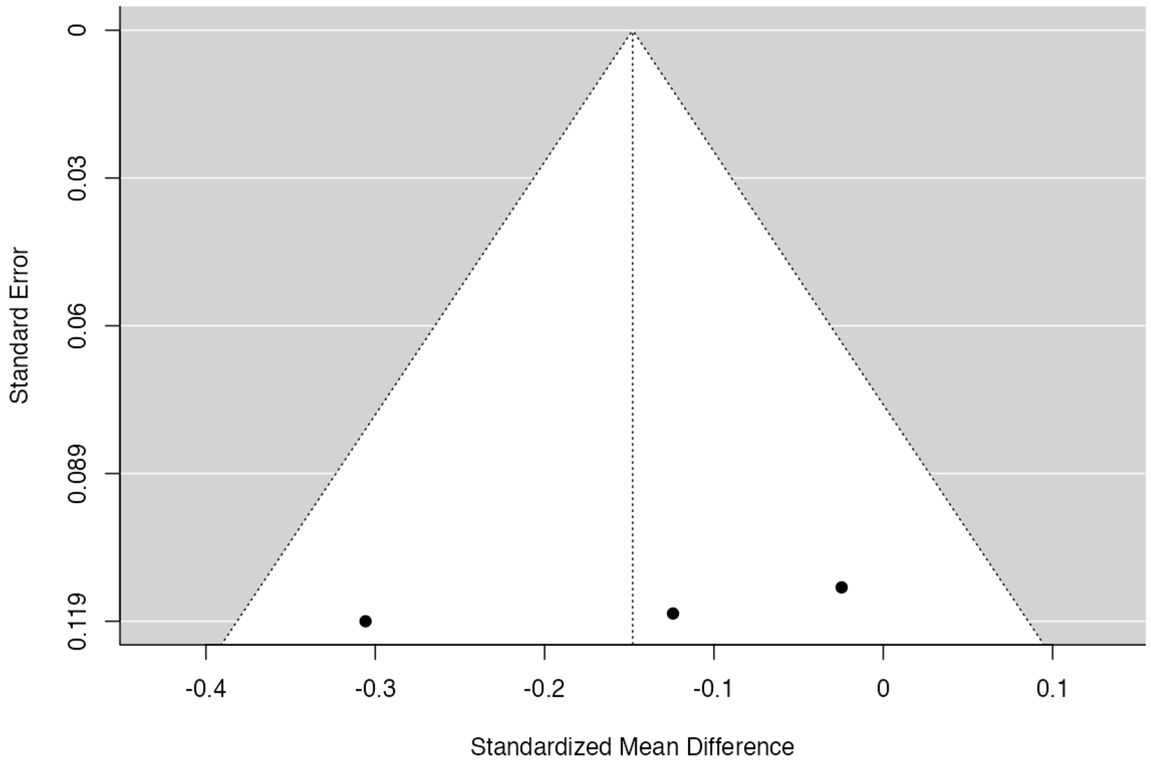

Supplementary Material I. Subgroup/Moderator Analysis – Intervention Provided to Parent/Caregiver(s)

Mean Differences (n, M, SD)

Random-Effects Model (k = 6)

	Estimate	se	Z	p	CI Lower Bound	CI Upper Bound
Intercept	-0.153	0.298	-0.514	0.607	-0.737	0.431
	.	.	.	.	.	.

Note. Tau^2 Estimator: Hedges

Heterogeneity Statistics

Tau	Tau^2	I^2	H^2	R^2	df	Q	p
0.679	0.4614 (SE= 0.3528)	95.63%	22.869	.	5.000	43.030	< .001

Forest Plot

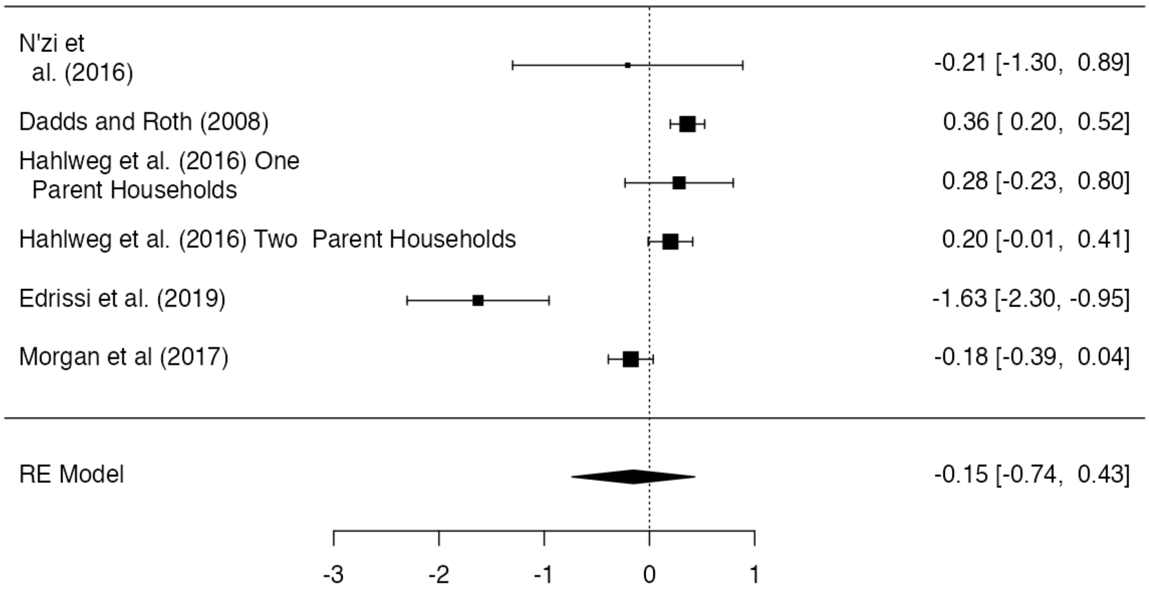

Publication Bias Assessment

Test Name	value	p
Fail-Safe N	0.000	0.412
Begg and Mazumdar Rank Correlation	-0.600	0.136
Egger's Regression	-0.979	0.327
Trim and Fill Number of Studies	0.000	.

Note. Fail-safe N Calculation Using the Rosenthal Approach

Funnel Plot

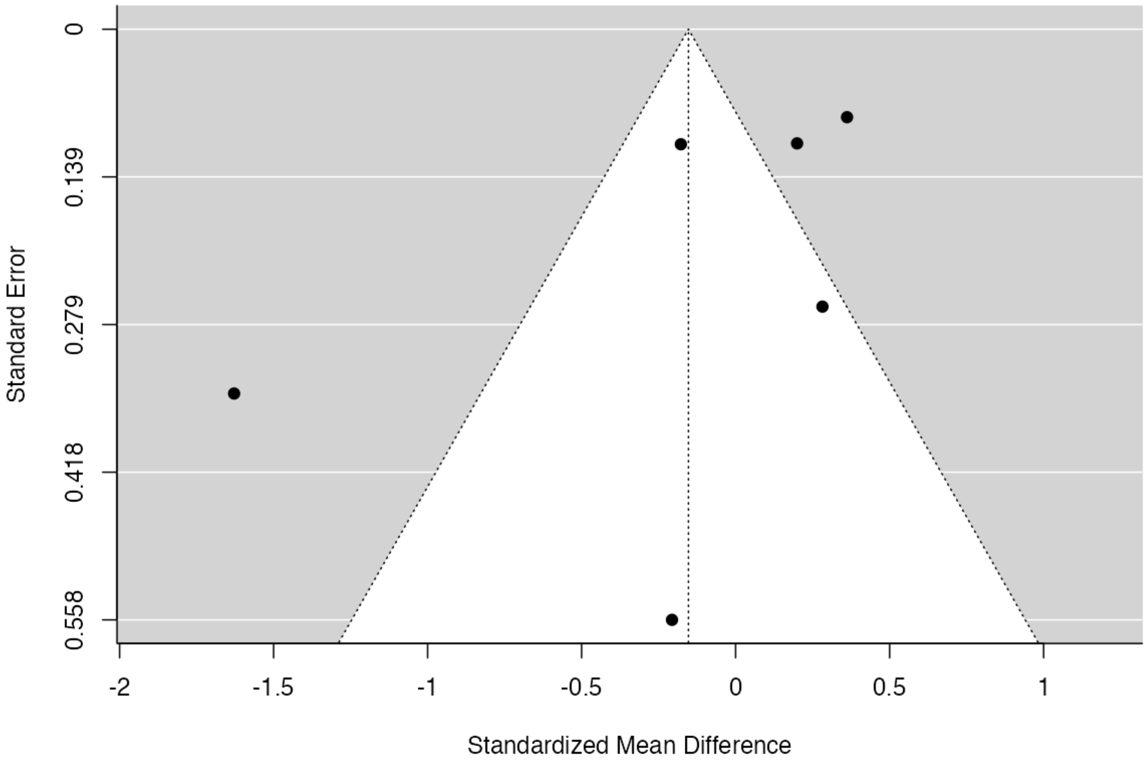
0
0.139
0.279
0.418
0.558
Standard Error
-2
-1.5
-1
-0.5
0
0.5
1
Standardized Mean Difference
